# Speed-driven transitions between discrete and rhythmic dynamics in walking revealed by kinematic smoothness and muscle synergies

**DOI:** 10.1016/j.isci.2026.117779

**Published:** 2026-10-07

**Authors:** Giulia Panconi, Diego Minciacchi, Riccardo Bravi, Nadia Dominici

**Affiliations:** 1 Department of Experimental and Clinical Medicine, University of Florence, Florence, Italy; 2 Department of Human Movement Sciences, Faculty of Behavioural and Movement Sciences, Vrije Universiteit Amsterdam, Amsterdam, the Netherlands; 3 Amsterdam Movement Sciences, Rehabilitation & Development, Amsterdam, the Netherlands; 4 Institute for Brain and Behaviour Amsterdam, Vrije Universiteit Amsterdam, Amsterdam, the Netherlands

**Keywords:** locomotion, walking speed, motor primitives, discrete movement, rhythmic movement, kinematic smoothness, muscle synergies, sEMG

## Abstract

Humans control movement through motor primitives that generate discrete and rhythmic actions. We investigated whether and how speed may drive a transition between discrete and rhythmic organization in walking, and whether muscle synergy changes are associated with kinematic shifts. Eighteen healthy adults walked on a treadmill during incremental and decremental trials (0.5–5 km/h in 0.5 km/h steps). Kinematics and bilateral lower-limb EMG were recorded. Smoothness was quantified using log dimensionless jerk (LDJ) and spectral arc length (SPARC). Both metrics indicated lower smoothness at low speeds and progressively stabilized as speed increased, with a transition region around 3–3.5 km/h showing inter-individual variability. In parallel, EMG synergies showed speed-dependent increases in dimensionality (2→3→4), consistent with module merging at slower speeds. Overall, these findings reveal coordinated kinematic and neuromuscular shifts with speed, indicating a transition from a discrete-dominated regime at low speeds toward a more stable rhythmic pattern at higher speeds.

## Highlights

• Walking speed acts as a control parameter shifting gait from discrete to rhythmic
• Gait smoothness drops at low speeds, consistent with discrete-like control
• Both kinematic smoothness metrics reveal a transition window near 3–3.5 km/h
• Lower walking speeds reduce muscle synergies from four to two via module merging

## Graphical abstract

**Figure:**
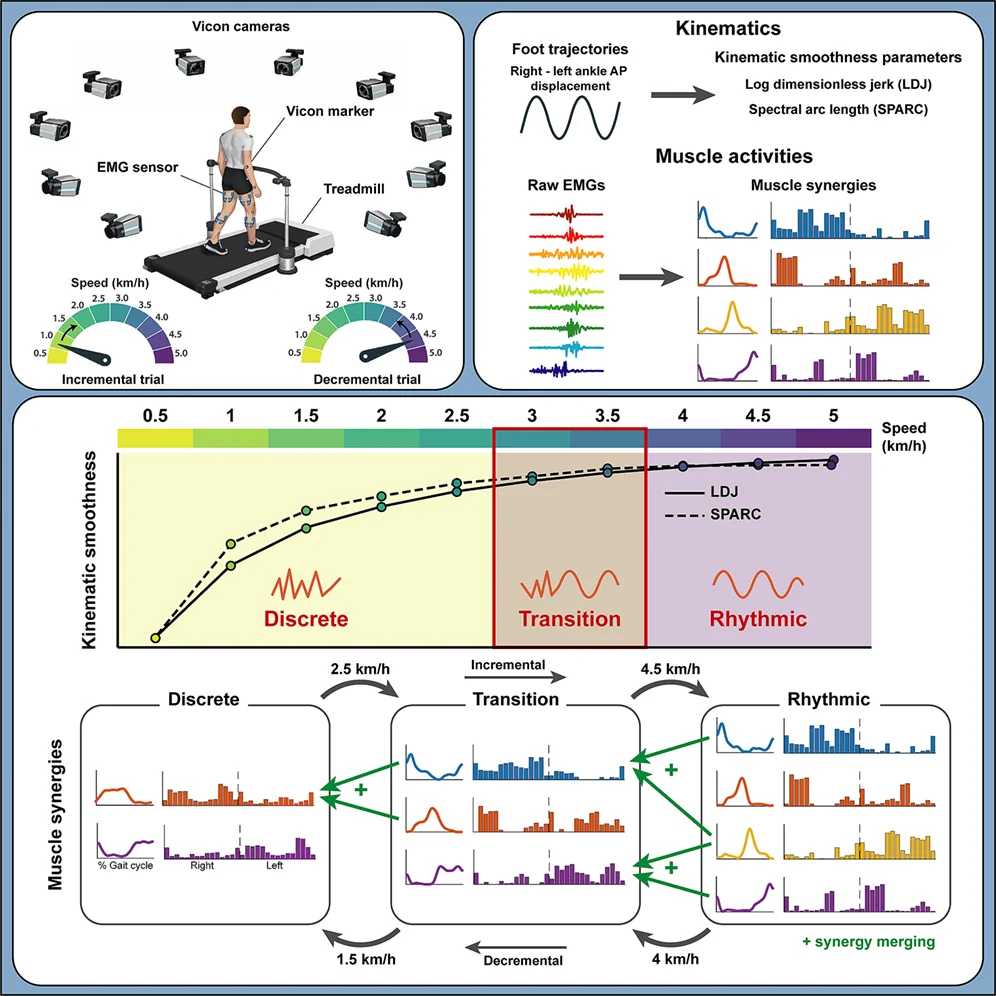

## Teaser

natural sciences; biological sciences; neuroscience; systems neuroscience

## Introduction

Human motor control has been suggested to emerge from a set of fundamental building blocks known as motor primitives.^1^^,^^2^^,^^3^^,^^4^^,^^5^^,^^6^^,^^7^^,^^8^ Within this framework, primitives provide a model of how the central nervous system generates and controls motor behavior and, at the end-effector level, give rise to discrete and rhythmic movements.^5^^,^^9^ Discrete movements are modeled as point-attractor dynamics (or submovements) and are characterized by transitions between distinct stable postural endpoints, whereas rhythmic movements are modeled as limit-cycle dynamics that produce periodic oscillations with stable phase relationships.^5^^,^^9^^,^^10^^,^^11^

Multiple lines of evidence support the theory that discrete and rhythmic movements recruit partly distinct neural networks. Neuroimaging studies have shown that discrete actions engage broader bilateral and non-primary motor areas relative to rhythmic ones, implicating partly separable cortical and cerebellar networks.^12^^,^^13^ At the behavioral level, motor learning studies have shown asymmetric transfer and reduced interference between discrete and rhythmic contexts, consistent with partly separable motor mechanisms.^14^^,^^15^ This distinction is also reflected in clinical populations, where rhythmic arm movements appear less impaired than discrete ones following stroke.^16^

This evidence does not imply a rigid dichotomy between movement classes. Research on upper-limb control has shown that producing very slow rhythmic movements is challenging and may fragment into a sequence of discrete submovements, suggesting that the motor system can shift flexibly between these two modes as task constraints change.^6^^,^^17^ Although most empirical evidence supporting the discrete-rhythmic framework derives from upper-limb movements, dynamic primitives are assumed to reflect general organizational principles of the central nervous system, and therefore similar transitions should in principle be observable across different effector systems.^5^^,^^9^ Locomotion is a particularly compelling case: unlike reaching, which is discrete by default, walking is prototypically rhythmic, so evidence of discrete-like organization in walking is especially informative where the framework is least expected. Slow walking is a natural setting in which to observe this. At very slow speeds, locomotor coordination may destabilize and fragment into discrete-like submovements, mirroring what has been reported for the upper limb. Slow walking is also common in aging and clinical populations,^18^^,^^19^ precisely the conditions in which rhythmic locomotor organization may be compromised, making these regime shifts significant to study.

Walking speed has been proposed as a control parameter triggering changes in coordination patterns consistent with phase-transition-like behavior.^20^^,^^21^^,^^22^ If this is the case, transitions between locomotor regimes may exhibit properties analogous to other phase transitions. The walk-to-run transition classically exhibits hysteresis, occurring at a higher speed when speed is increasing than when decreasing,^20^^,^^23^ and an analogous direction-dependence has been reported within walking for control of the center of mass.^24^

Tracking such transitions within walking, however, first requires a way to characterize the discrete and rhythmic dynamics of gait at each speed. In a recent study, Moura Coelho et al. showed that kinematic smoothness measures can serve as a proxy for distinguishing discrete-like from rhythmic-like walking patterns, with very slow walking exhibiting stronger discrete-like features and faster walking reflecting a more stable rhythmic organization.^25^ These findings suggest that locomotion may not be uniformly rhythmic across speeds, but rather operate within a range of regimes whose expression depends on task constraints. Although such speed-dependent discrete rhythmic regime shifts may occur in locomotion, the mechanisms driving them remain poorly understood.

Despite the increasing evidence, key questions remain regarding how discrete-rhythmic regime shifts evolve across a range of walking speeds and whether consistent patterns emerge across individuals. Furthermore, the existing evidence within the discrete-rhythmic primitives literature remains predominantly kinematic, leaving open whether kinematic regime shifts are accompanied by systematic reorganization of neuromuscular activity. Since smoothness is an end-effector readout, it cannot by itself establish whether a kinematic regime shift reflects a reorganization of locomotor control or merely the biomechanical consequences of reduced walking speed. Resolving this distinction therefore requires converging evidence from the neuromuscular level.

Muscle synergy analysis provides a strong framework to characterize the modular organization of locomotor muscle activity, where groups of muscles are co-activated in coordinated patterns that can be flexibly recruited across task conditions.^26^^,^^27^ EMG-based studies have shown that distinct spinal locomotor networks can be differentially recruited across locomotor modes and that muscle activation patterns may reorganize with speed even within the same locomotor mode,^28^ raising the possibility that transitions observed at the kinematic level may reflect underlying changes in the organization of neuromuscular control. Because muscle synergies provide an indirect representation of the dimensionality and structure of coordinated muscle activation rather than the mechanical output of the limb, a speed-dependent change in synergy organization would support the interpretation that a kinematic regime shift extends to the level of neuromuscular control.

Smoothness and muscle synergy analyses provide complementary perspectives on motor control. Consistent changes across both measures would therefore offer stronger evidence for a genuine reorganization of locomotor control than either measure alone. This distinction is particularly relevant in populations with reduced walking speed, where separating real control adaptations from the kinematic consequences of slower movement is essential for interpreting gait changes and guiding interventions.

Accordingly, this study aims to characterize the speed-dependent transition between discrete-like and rhythmic-like locomotor patterns and determine whether kinematic regime shifts are accompanied by systematic EMG reorganization assessed through changes in muscle synergies, thereby linking changes in movement dynamics to their underlying control mechanisms.

## Results

### Speed-dependent changes in kinematic smoothness

Across the incremental and decremental trials (Figure 1), both smoothness metrics showed a clear dependence on walking speed, with greater variability at low walking speeds and progressively more stable values at the highest speeds (Figures 2 and 3).

**Figure 1:**
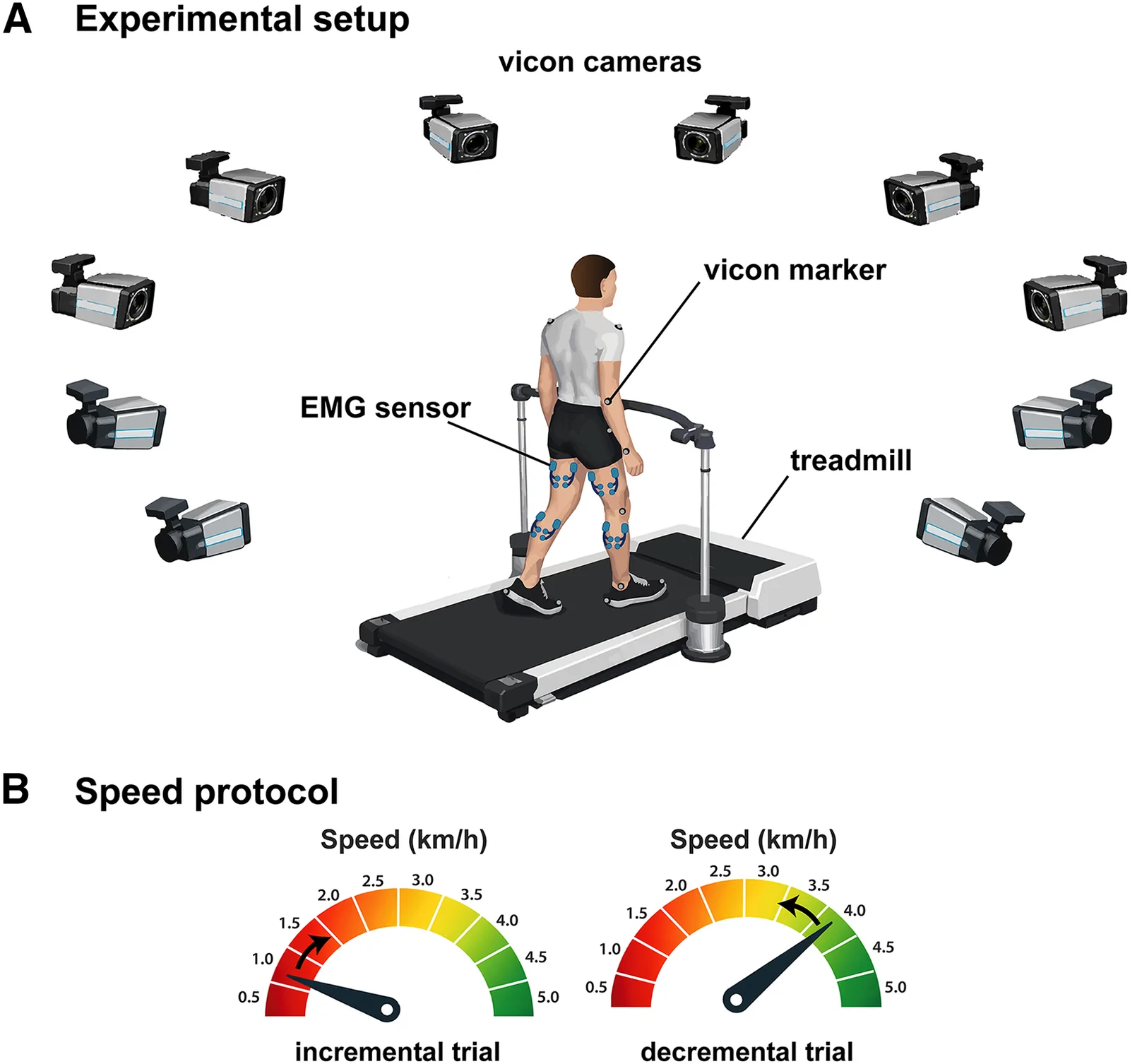
Experimental setup and walking speed protocol (A) Participants walked on a treadmill while kinematics were recorded with a 10-camera Vicon motion capture system. Reflective markers were placed bilaterally on anatomical landmarks (upper limb, pelvis, and lower limb), and EMG activity was recorded bilaterally: from lower-limb muscles. (B) Participants performed an incremental trial with speed increased from 0.5 to 5.0 km/h in 0.5 km/h steps, and a decremental trial with speed decreased from 5.0 to 0.5 km/h in 0.5 km/h steps.

**Figure 2:**
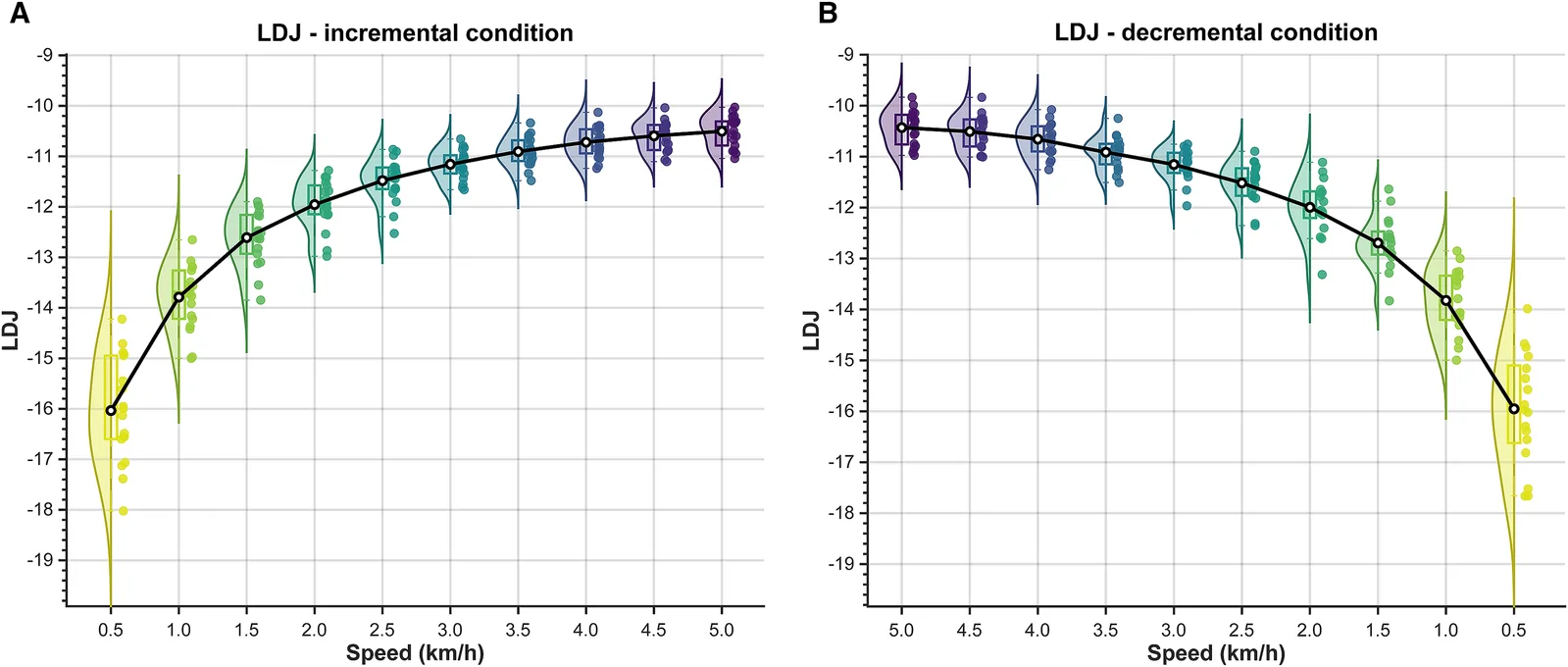
Speed-dependent changes in movement smoothness quantified by LDJ (A) Distribution of LDJ values computed for each speed segment for the incremental condition. (B) Distribution of LDJ values in the decremental condition. In both panels, colored points represent each participant’s mean at that speed segment, and the dots connected by a black line indicate the group mean across participants. LDJ, log dimensionless jerk.

**Figure 3:**
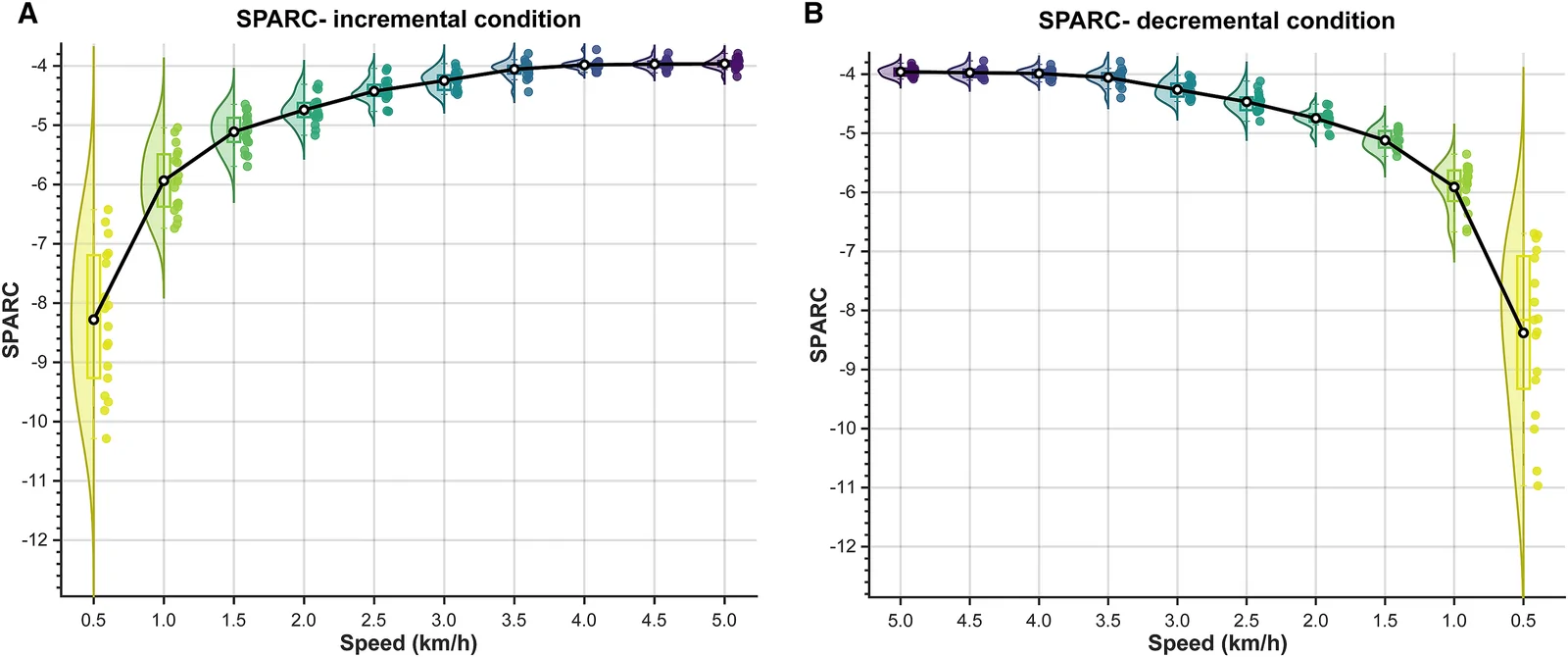
Speed-dependent changes in movement smoothness quantified by SPARC (A) Distribution of SPARC values computed for each speed segment for the incremental condition. (B) Distribution of SPARC values in the decremental condition. In both panels, colored points represent each participant’s mean at that speed segment, and the black dot connected by a black line indicates the group mean across participants. SPARC, spectral arc length.

Log dimensionless jerk (LDJ) values increased (i.e., became less negative) with speed in both conditions, with mean subject’s values increasing from −16.03 ± 1.14 and −15.95 ± 1.20 at 0.5 km/h (incremental and decremental, respectively) to −10.50 ± 0.34 and −10.43 ± 0.36 at 5 km/h (Figure 2).

A similar pattern was observed for spectral arc length (SPARC), with values becoming progressively less negative as speed increased, from −8.28 ± 1.55 and −8.38 ± 1.71 at 0.5 km/h (incremental and decremental, respectively) to −3.96 ± 0.09 and −3.96 ± 0.08 at 5 km/h.

Within-trial repeated-measures ANOVA revealed a significant effect of speed on LDJ across subjects (*p* < 0.001) (Figure 4; see Table S4). Pairwise post hoc comparisons revealed highly consistent LDJ differences at low-to-moderate speeds: from 0.5 to 2.5 km/h, comparisons involving lower-speed segments showed high consistency across participants (typically ≥89%–100%). This consistency dropped to a moderate range around 3–3.5 km/h (approximately 50%–80%, depending on the specific contrast), and became low at the highest speeds (4–4.5 km/h), where only a small fraction of subjects showed significant differences (0%–11%) between high-speed segments (Figure 4; see Table S7).

**Figure 4:**
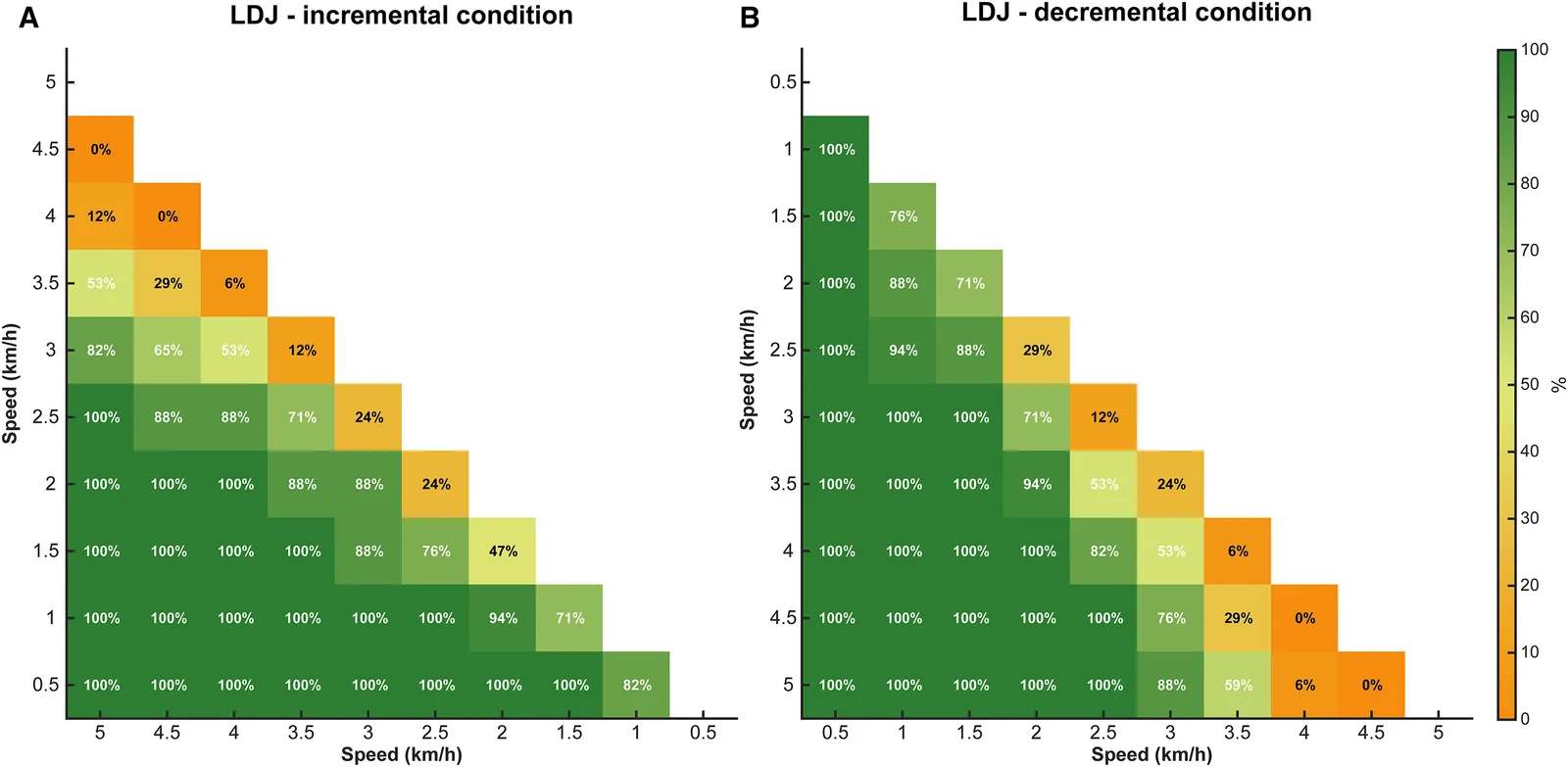
Heatmaps showing the percentage of participants with significant LDJ pairwise differences between speed segments In incremental (A) and decremental (B) conditions, the percentage of participants showing a statistically significant difference in LDJ between each pair of walking speeds (0.5–5.0 km/h in 0.5 km/h steps). Each cell reports the % of participants with a significant pairwise contrast for the corresponding speed combination (axes), with color indicating the proportion (orange = lower, green = higher). Higher percentages indicate speed pairs that more consistently elicited LDJ changes across individuals. LDJ, log dimensionless jerk.

Within-trial repeated-measures ANOVA showed a consistent significant effect of speed on SPARC across participants (*p* < 0.001) (Figure 5). Pairwise post hoc comparisons revealed highly consistent SPARC differences at low-to-moderate speeds: from 0.5 to 2.5 km/h, comparisons involving lower-speed segments showed high consistency across participants (≥89%). This consistency dropped to a moderate-to-high range around 3 km/h (approximately 80%) and further decreased to low-to-moderate levels around 3.5 km/h (approximately 20%–30%). At the highest speeds (4–5 km/h), agreement was very low, with no participants showing significant differences between segments (Figure 5; see Table S7).

**Figure 5:**
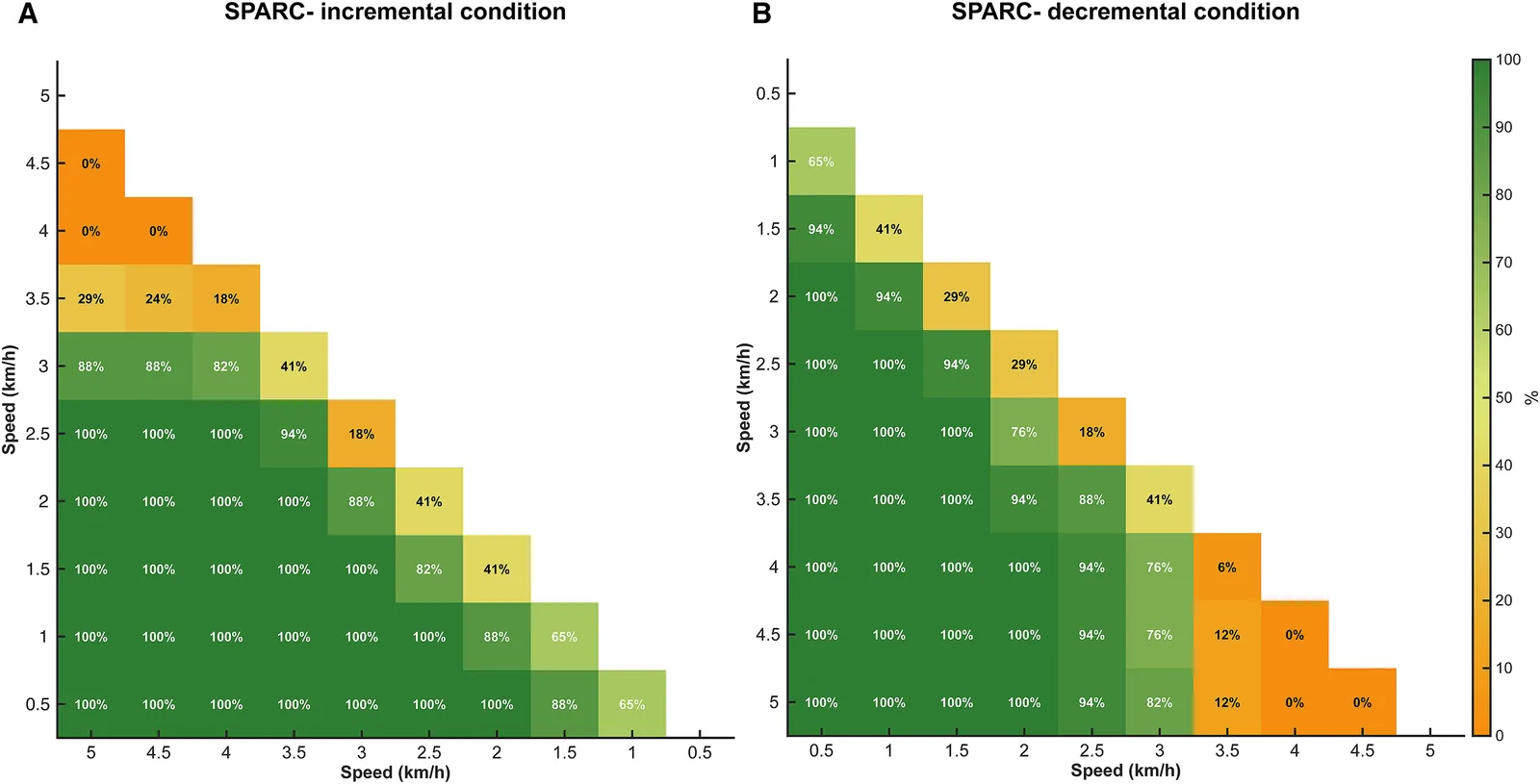
Heatmaps showing the percentage of participants with significant SPARC pairwise differences between speed segments In incremental (A) and decremental (B) conditions, the percentage of participants showing a statistically significant difference in SPARC between each pair of walking speeds (0.5–5.0 km/h in 0.5 km/h steps). Each cell reports the % of participants with a significant pairwise contrast for the corresponding speed combination (axes), with color indicating the proportion (orange = lower, green = higher). Higher percentages indicate speed pairs that more consistently elicited SPARC changes across individuals. SPARC: spectral arc length.

In parallel with these smoothness changes, participants showed speed-dependent modulation of classical gait parameters in both conditions (see Tables S2 and S3). As walking speed increased, stride length and cadence increased progressively (in the incremental condition, from 56.4 ± 10.5 to 138.1 ± 5.9 cm and from 31.2 ± 4.6 to 111.4 ± 4.7 steps/min, respectively), while stride time shortened (from 4.00 ± 0.86 to 1.08 ± 0.04 s). The relative stance duration decreased (from 83.4 ± 2.1% to 66.5 ± 0.7% of the stride), with a corresponding increase in the swing phase, and the double-support phase decreased steadily with speed.

### Speed-dependent modulation of muscle synergy dimensionality

Across speeds, the number of muscle synergies identified at the participant level showed a clear speed-dependent modulation in both conditions (mean ± SD across participants) (Figure 6). A one-factor repeated measures ANOVA revealed a significant main effect of speed on the number of extracted synergies in both the incremental and decremental conditions (see Table S5).

**Figure 6:**
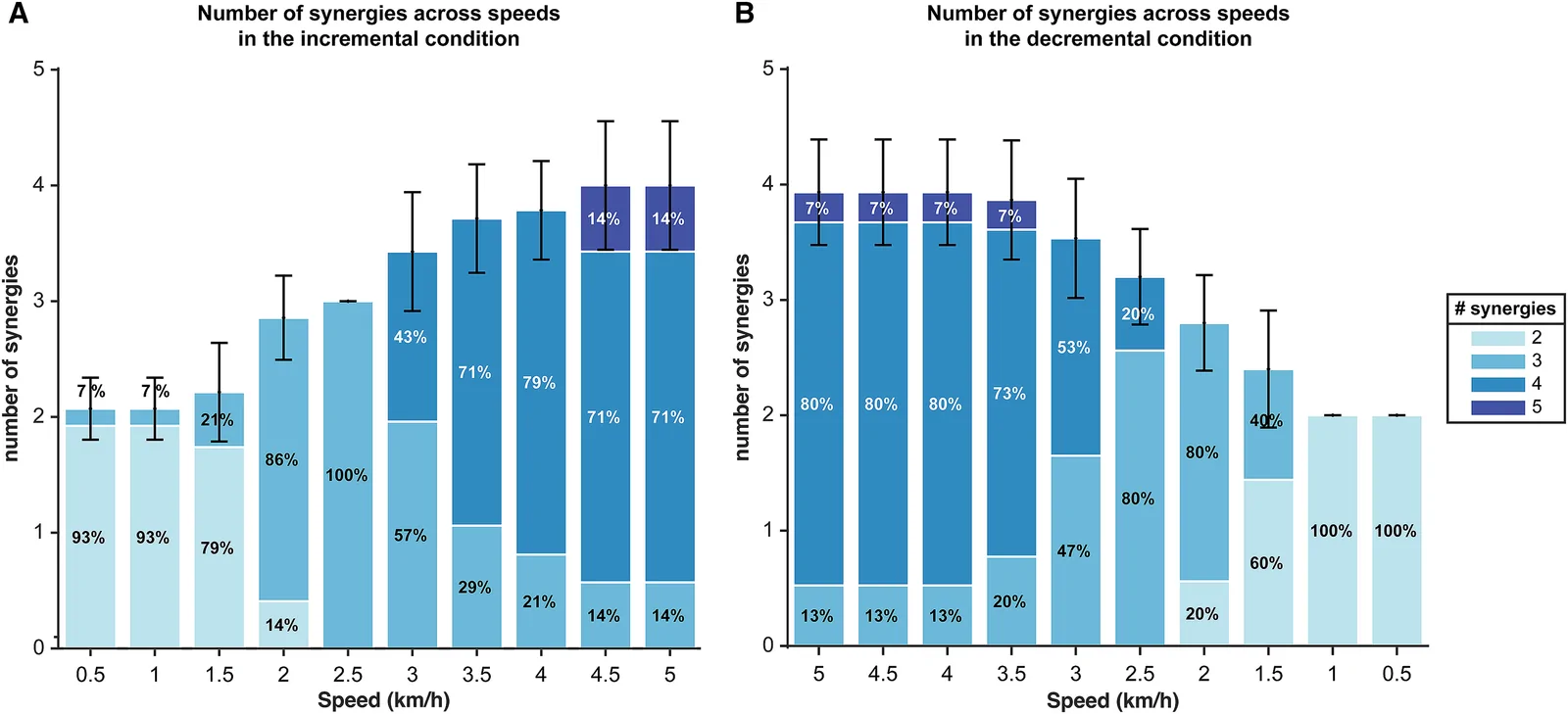
Speed-dependent distribution of the number of muscle synergies Stacked bar plots show, for each speed-segment, the percentage of participants whose EMG decomposition resulted in a given number of synergies (2–5) during the incremental (A) and decremental (B) protocols. Error bars represent the between-participant standard deviation of the mean synergy number at each speed.

In the incremental condition, the mean number of synergies increased with speed, from 2 synergies at 0.5–1.5 km/h to 3 synergies at 2–3 km/h, reaching 4 synergies from 3 km/h onward and stabilizing around 4 synergies at 4.5–5 km/h (Figure 6A). In the decremental condition, the mean number of synergies was highest at fast walking (4 synergies at 5–3 km/h) and progressively decreased as speed was reduced, reaching ∼3 synergies at 3 km/h, and ∼2 synergies at 1.5–0.5 km/h (Figure 6B).

Pairwise post-hoc comparisons (Holm-adjusted) indicated a stabilization of the number of muscle synergies at higher walking speeds in both conditions (see Tables S8 and S9). In the incremental condition, no differences were observed between 5.0 km/h and 4.5, 4.0, or 3.5 km/h (all *p* ≥ 0.441). In contrast, significant differences emerged when comparing the high-speed segments with 3.0 km/h (5.0 vs. 3.0 km/h: *p* = 0.018; 4.5 vs. 3.0 km/h: *p* = 0.018) (Figure 7A). In the decremental condition, the number of synergies at 5.0 km/h did not differ from 4.5, 4.0, 3.5, or 3.0 km/h (all *p* ≥ 0.137). The first significant difference appeared at 2.5 km/h where the number of synergies differed from high-speed segments (5.0, 4.5, and 4.0 vs. 2.5 km/h: *p* = 0.001) (Figure 7B).

**Figure 7:**
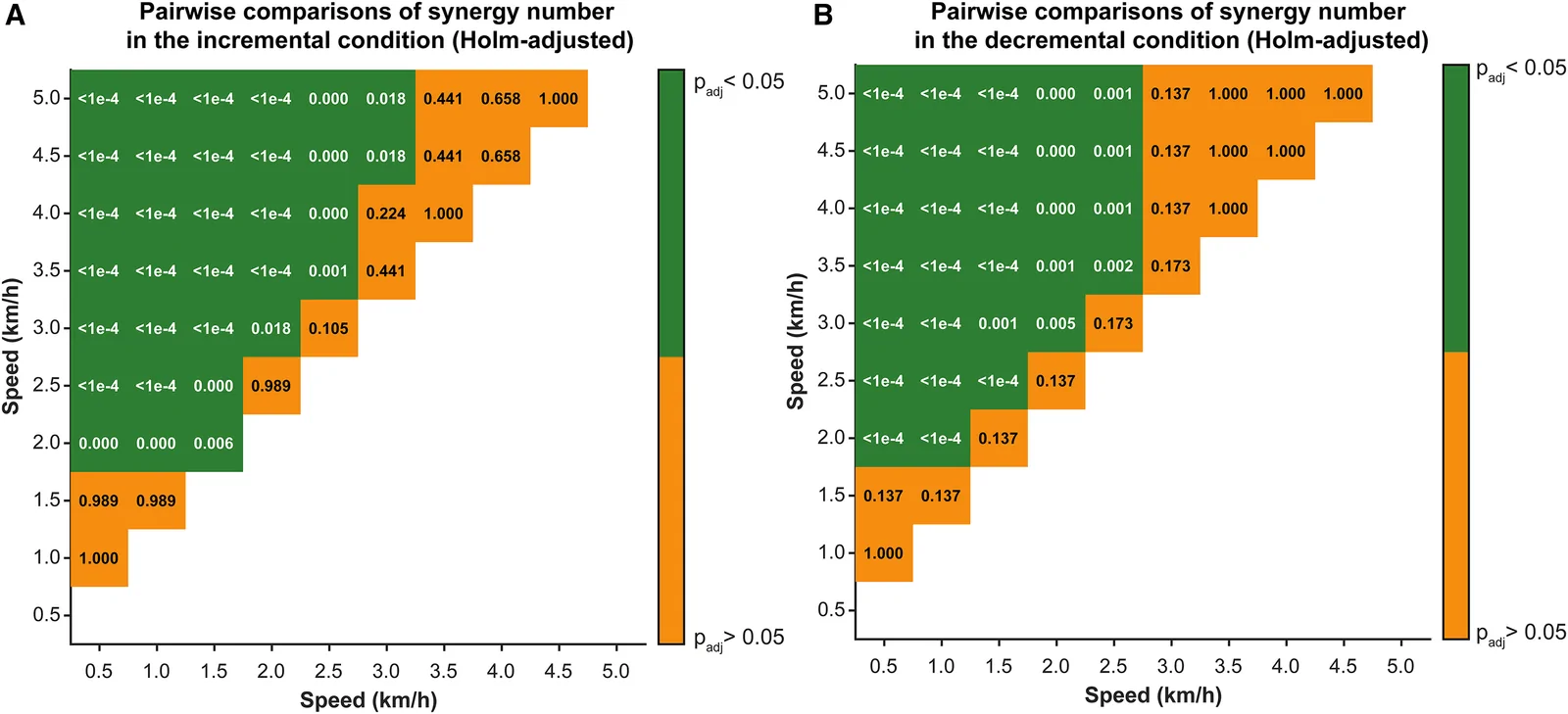
Heatmaps showing post hoc pairwise differences in synergy number across walking speeds In incremental (A) and decremental (B) conditions, each cell reports the Holm-adjusted *p* value for the pairwise comparison between the two speed segments (km/h); green indicates significant differences (p_adj_ < 0.05), and orange indicates non-significant comparisons (p_adj_ ≥ 0.05). In both conditions, comparisons among the highest speeds show non-significant differences, whereas significant differences emerge when contrasting high-speed segments with lower-speed segments from 3 km/h in the incremental condition and 2.5 km/h in the decremental condition.

At the group level, the EMG signals used for synergy extraction were averaged across participants within each speed-segment and condition (incremental and decremental). Variance accounted for (VAF) curves were then computed from the resulting group-averaged EMG matrices for each speed-segment as a function of the number of extracted synergies in order to determine the minimum number of synergies meeting the VAF criterion (Figure 8). The curves show that higher walking speeds required a greater number of synergies to reach the VAF criterion.

**Figure 8:**
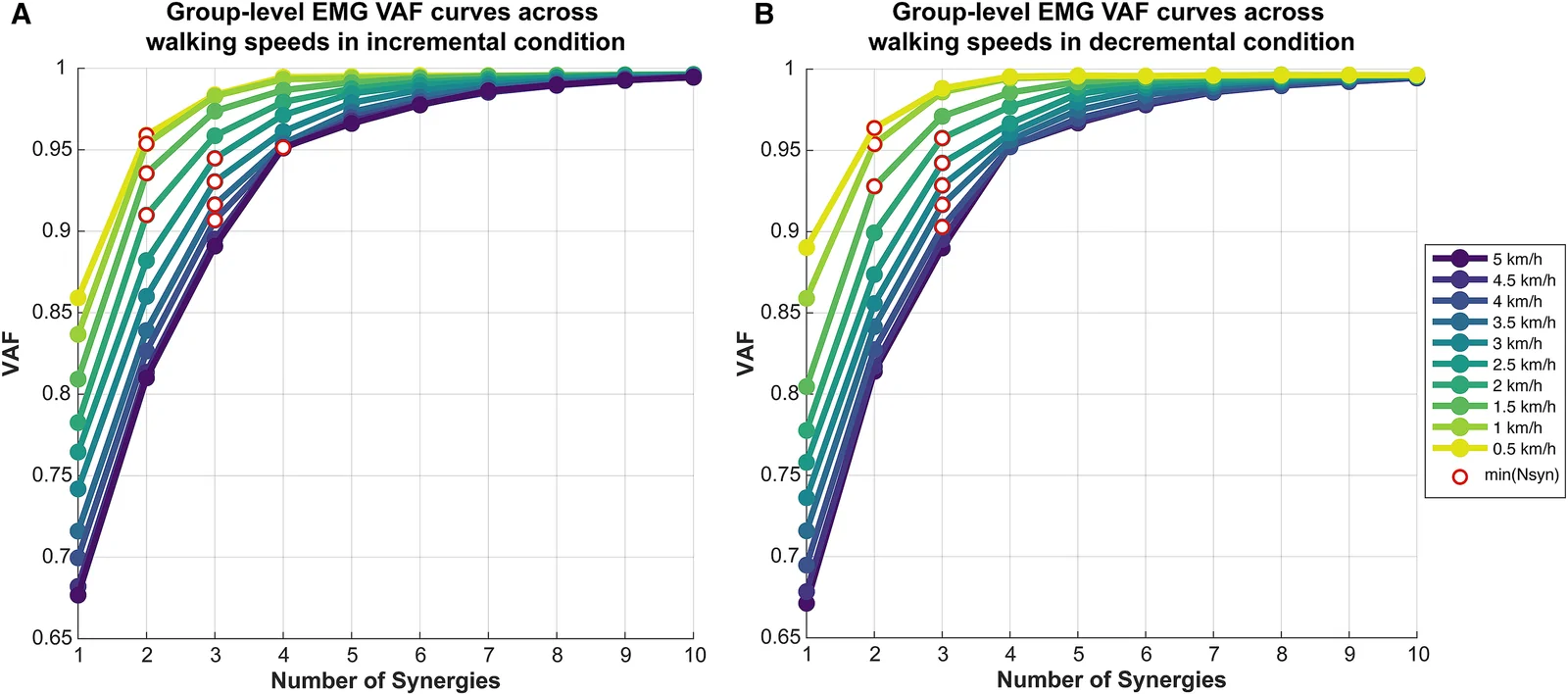
Group-level EMG VAF curves across walking speeds VAF of the reconstructed EMG signal in the incremental (A) and decremental (B) conditions is plotted as a function of the number of extracted muscle synergies (1–10) for each speed segment (0.5–5 km/h; speed color-coded). Open red circles indicate the selected minimal number of synergies (min(*N*syn)) at each speed according to the adopted VAF criterion (VAF ≥ 0.90).

The number of synergies required to explain EMG variance showed a clear dependence on walking speed in both conditions (Figures 9 and 10). In the incremental condition, low speeds (0.5–2 km/h) were adequately described by two synergies. From 2.5 km/h onward, an additional synergy emerged, yielding three synergies across intermediate speeds. At the highest speeds (4.5–5 km/h), a fourth synergy was identified (Figure 9). In the decremental condition, four synergies were expressed at the highest speeds (5–4.5 km/h), after which the solution reduced to three synergies from 4 to 2 km/h. At the lowest speeds (≤1.5 km/h), the average modular organization further reduced to two synergies (Figure 10).

**Figure 9:**
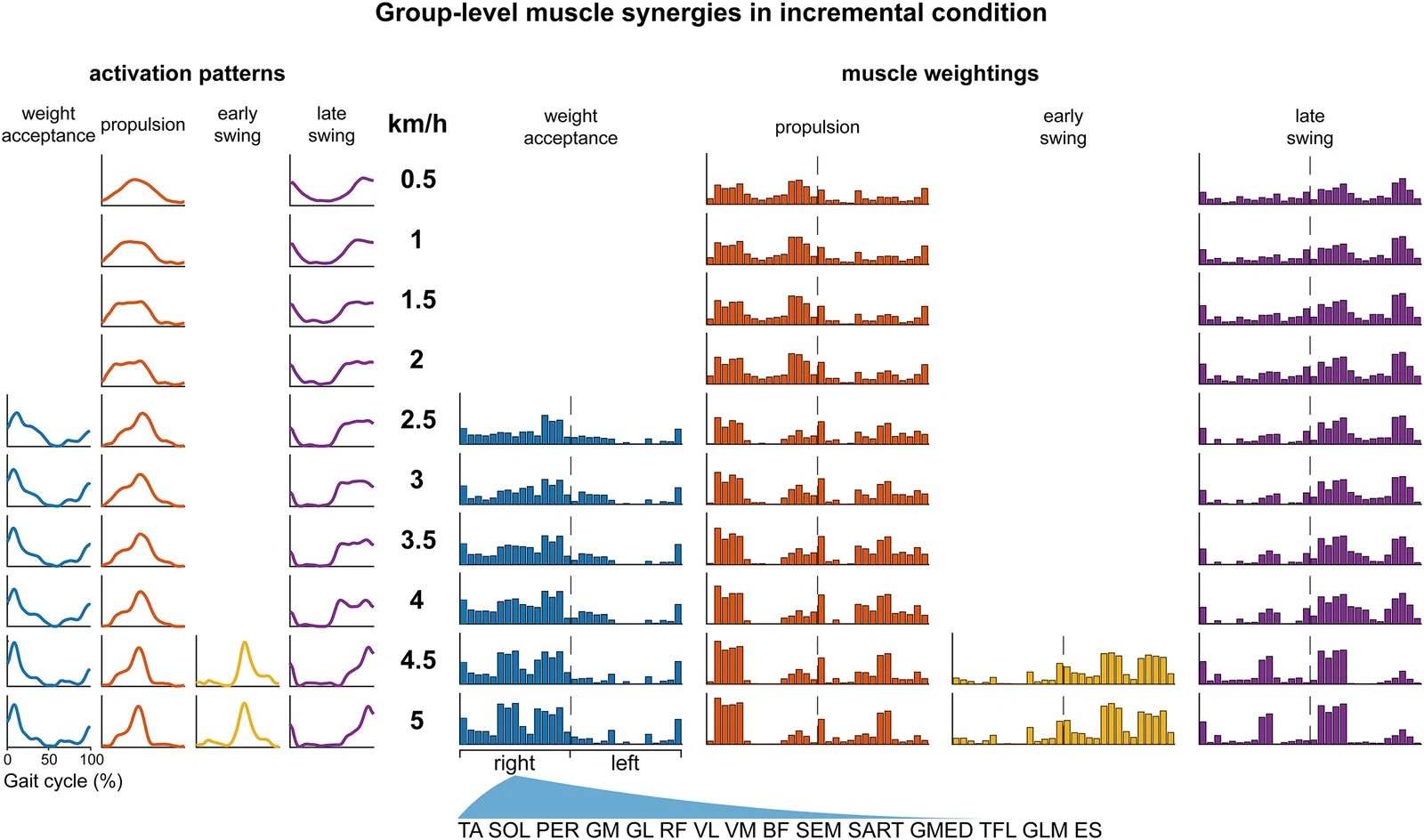
Group-level muscle synergies across speeds in the incremental condition For each speed segment (0.5–5.0 km/h; rows), the figure shows the extracted synergy activation patterns (H; line plots on the left) and the corresponding muscle weightings (W; bar plot on the right). Activation patterns are time-normalized to the gait cycle (0%–100%) and organized according to the functional phase of the gait cycle (weight acceptance, propulsion, early swing, and late swing). These functional phase labels denote the dominant functional epoch of each activation component and are intended to be descriptive; this organization corresponds to the canonical set of activation components reported across human walking studies.^4^^,^^29^^,^^30^^,^^31^ Bar plots show normalized muscle weights, with dashed vertical lines separating right- and left-side muscle groups. TA, tibialis anterior; SOL, soleus; PER, peroneus longus; GM, gastrocnemius medialis; GL, gastrocnemius lateralis; RF, rectus femoris; VL, vastus lateralis; VM, vastus medialis; BF, biceps femoris; SEM, semitendinosus; SART, sartorius; GMED, gluteus medius; TFL, tensor fasciae latae; GLM, gluteus maximus; ES, erector spinae.

**Figure 10:**
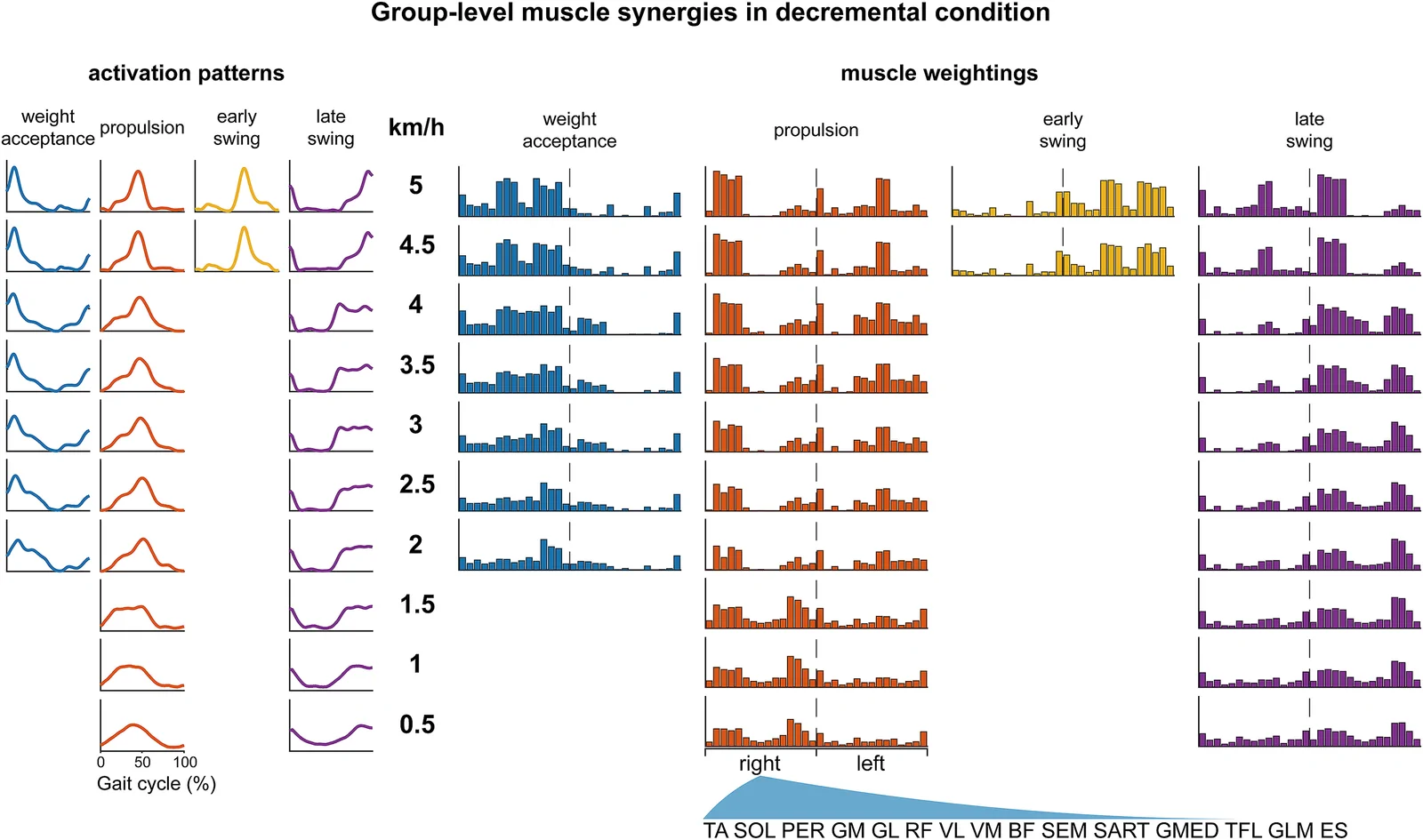
Group-level muscle synergies across speeds in the decremental condition For each speed segment (5–0.5 km/h; rows), the figure shows the extracted synergy activation patterns (H; line plots on the left) and the corresponding muscle weightings (W; bar plot on the right). Activation patterns are time-normalized to the gait cycle (0%–100%) and organized according to the functional phase of the gait cycle (weight acceptance, propulsion, early swing, and late swing). These functional phase labels denote the dominant functional epoch of each activation component and are intended to be descriptive; this organization corresponds to the canonical set of activation components reported across human walking studies.^4^^,^^29^^,^^30^^,^^31^ Bar plots show normalized muscle weights for the recorded muscles (labels in the bottom), with dashed vertical lines separating right- and left-side muscle groups. TA, tibialis anterior; SOL, soleus; PER, peroneus longus; GM, gastrocnemius medialis; GL, gastrocnemius lateralis; RF, rectus femoris; VL, vastus lateralis; VM, vastus medialis; BF, biceps femoris; SEM, semitendinosus; SART, sartorius; GMED, gluteus medius; TFL, tensor fasciae latae; GLM, gluteus maximus; ES, erector spinae.

### Within-subject correlation between smoothness and synergy dimensionality

Within each participant, kinematic smoothness metrics and the number of extracted synergies were positively correlated across speed segments (see Table S6; Figure S1). In the incremental condition, the within-subject Spearman correlation was significant in all participants for both metrics (median ρ = 0.92, IQR 0.92–0.94). The decremental condition yielded essentially identical results (significant in all participants; median ρ = 0.92, IQR 0.88–0.93), and combining both conditions preserved the relationship (median ρ = 0.91; significant in all participants). Thus, as walking became smoother with increasing speed, the number of extracted synergies increased correspondingly in every participant, irrespective of ramp direction.

### Muscle synergy merging across walking speeds

Merging analysis revealed evidence of module merging across adjacent faster-to-slower speed-segment pairs, particularly at transitions where the number of synergies changed (Figure 11). In the incremental condition, merging was observed at 2 km/h ← 2.5 km/h for target synergy 1, reconstructed from synergies 1 (m_ik_ = 0.58) and 2 (m_ik_ = 0.54) (cosine = 0.99). A stronger merging pattern emerged at 4 km/h ← 4.5 km/h, where two target synergies were classified as merged: synergy 1 (cosine = 0.99), reconstructed from 1 to 3 (m_ik_ = 0.88, m_ik_ = 0.23) and synergy 3 (cosine = 0.98) from 3 to 4 (m_ik_ = 0.60, m_ik_ = 0.59) (Figure 11A). In the decremental condition, merging was detected at 4 km/h ← 4.5 km/h for the same two targets (target 1 (cosine = 0.99) reconstructed from synergies 1 and 3 (m_ik_ = 0.83, m_ik_ = 0.27) and target 3 (cosine = 0.97) from synergies 3 and 4 (m_ik_ = 0.57, m_ik_ = 0.62), and at 1.5 km/h ← 2.0 km/h for target 1 (cosine = 0.99) reconstructed from synergies 1 and 2 (m_ik_ = 0.56, m_ik_ = 0.59) (Figure 11B).

**Figure 11:**
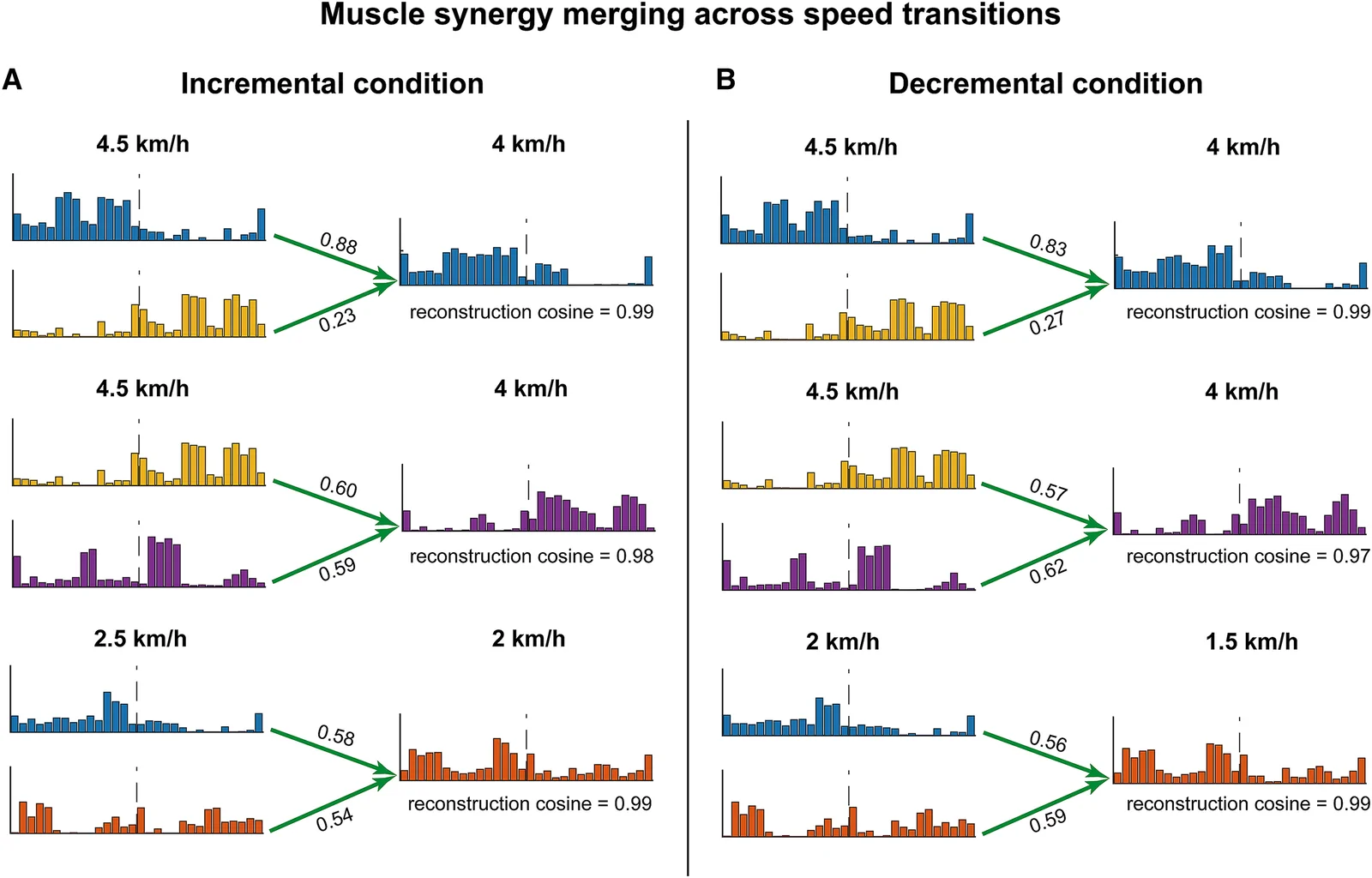
Examples of muscle synergy merging across speed transitions In incremental (A) and decremental (B) conditions, each row shows the colored bar plots of muscle weightings (W) of the contributing synergies from the adjacent faster speed segment on the left, and the reconstructed target synergy in the slower segment on the right. Green arrows indicate the contributing synergies used for reconstruction, and the values next to the arrows report the corresponding non-negative reconstruction coefficients (m_ik_). The overall reconstruction cosine similarity for each target synergy is reported below the right-hand figure. Examples illustrate merging at 2.5→2.0 and 4.5→4.0 km/h in the incremental condition and at 4.5→4.0 and 2.0→1.5 km/h in the decremental condition.

## Discussion

Overall, the present results show that walking speed modulates the balance between discrete- and rhythmic-like components of locomotor control, with convergent changes in both the kinematic smoothness and the dimensionality of the underlying muscle synergies. Movement smoothness metrics highlighted a transition from a more discrete-like organization at low speeds toward a more rhythmic regime at higher speeds. Specifically, across both incremental and decremental trials, LDJ and SPARC increased with speed (i.e., became less negative), indicating progressively smoother movements, while the lowest speeds showed the most negative values, reflecting reduced smoothness. In parallel, the number of muscle synergies required to reconstruct EMG activity increased with speed, so that the kinematic smoothing of gait was accompanied by a richer modular organization of muscle activity. Within every participant, smoothness and synergy number were strongly and positively correlated across speed segments.

The reduced smoothness at low speeds is consistent with literature suggesting that rhythmic movements become less smooth when performed slowly, because they fragment into a sequence of discrete submovements; the reduced smoothness is the kinematic signature of this fragmentation.^6^^,^^10^ Within the dynamic-primitives framework: extremely slow walking may reflect a more discrete-like control regime, whereas at higher speeds smoothness stabilizes, suggesting the emergence of a more stable rhythmic organization.^6^^,^^10^ This speed-dependent shift in control was further supported by the between participant analysis, in which the agreement decreased most markedly in an intermediate speed band (∼3.0–3.5 km/h), indicating a transition window in which participants showed heterogeneous responses before converging at faster walking speeds. This band was descriptively identified from the cross-participant heatmaps (Figures 4 and 5) which consistently indicated a transition window around ∼3.0–3.5 km/h across LDJ and SPARC, and in both incremental and decremental conditions. These findings suggest that participants transition into a more stable rhythmic attractor at slightly different speeds, leading to reduced agreement within this intermediate range. At higher speeds, responses appeared to converge, suggesting stabilization within a predominantly rhythmic control regime.^1^^,^^5^^,^^17^

Furthermore, smoothness values showed higher between- and within-participant variability at the slowest speeds and stabilized with reduced variability at moderate-to-high speeds. This is consistent with evidence that slow walking is associated with increased kinematic variability and systematic speed-dependent changes in local dynamic stability (e.g., Lyapunov-based measures).^32^^,^^33^^,^^34^ These findings support a nonlinear speed-smoothness relationship in which small increases at very slow walking speeds are associated with comparatively large changes in movement organization, whereas at moderate-to-fast speeds smoothness approaches stabilization and adjacent speed steps become less discriminable.^32^

The present study further characterized muscle synergy organization to test whether the kinematic regime shift extended to the structure of neuromuscular control. Subject-level muscle synergies exhibited a clear dependence on walking speed in both incremental and decremental conditions. At low speeds, activity was typically reconstructed with two synergies, increasing to three synergies at intermediate speeds and to four synergies at higher speeds. This aligns with evidence that human walking can be represented by a limited set of modules whose dimensionality varies with speed.^28^ At first sight, this increase in synergy dimensionality may appear at odds with the reduced smoothness observed at slower walking speeds. However, these findings are not contradictory, as smoothness and muscle synergy dimensionality provide complementary descriptions of locomotor organization rather than alternative measures of the same phenomenon. Within the dynamic-primitives framework, low-speed walking fragments into discrete-like submovements, while the corresponding muscle activity is organized through fewer, merged modules. A plausible physiological explanation is the increasing dynamic demands associated with faster walking. Higher walking speeds require greater propulsive forces, tighter phase-specific timing, and increased balance and foot-clearance demands, which likely require a richer modular organization of muscle activity. Consistent with this interpretation, comparable low-dimensional, merged synergy structures are observed when gait is immature or clinically impaired: in early development, before mature locomotor modules differentiate,^4^ and in slowed, impaired walking after stroke, where healthy modules merge and coordination complexity is reduced.^30^^,^^35^^,^^36^^,^^37^

The asymmetry of transition points in synergy number between the incremental and decremental conditions is consistent with history dependence (hysteresis) in locomotor coordination. Within a dynamical-systems perspective, motor behavior settles into stable attractors whose stability is modulated by control parameters such as speed. Because the system’s current state depends on its recent history, the speed at which reorganization occurs may differ depending on whether walking speed is increasing or decreasing.^35^ This behavior is analogous to the hysteresis reported for the walk-run transition, where gait switching occurs at different speeds during acceleration and deceleration.^20^^,^^21^^,^^23^^,^^38^ In the present study, the higher-dimensional modular organization observed at faster speeds persisted to lower speeds during deceleration, suggesting that the neuromuscular system temporarily remains in the previously established coordination state before reorganizing into a lower-dimensional modular organization.^23^^,^^38^

By contrast, smoothness showed only minimal directional differences across walking speed, and the participant-consistency heatmaps delineated the same transition window in both conditions (Figures 4 and 5). Thus, the direction dependence was evident primarily in the neuromuscular measure, whereas both smoothness and muscle synergy organization provided convergent evidence for the same speed-dependent reorganization of locomotor control.

Group-averaged results reproduced the subject-level synergy patterns. Synergy-merging analyses indicated that shifts in synergy number were concentrated at the transition speeds and were consistent with module merging: when stepping from a higher to an adjacent lower speed, the lower-speed synergies could be reconstructed with very high similarity as non-negative linear combinations of the higher-speed synergies, consistent with merging rather than module elimination. Our results are in line with synergy-merging literature, where altered or lower-dimensional synergy sets can be explained as combinations of a higher-dimensional reference set.^36^^,^^37^^,^^39^^,^^40^

Overall, kinematic and EMG findings provided converging evidence of speed-dependent reorganization. At low speeds, walking was less smooth and more variable, and muscle activity was captured by a low-dimensional set of two synergies, consistent with a more discrete-like regime. Across an intermediate transition window, smoothness increased while an additional synergy emerged, and between-participant agreement dropped, indicating that participants shifted regimes at slightly different speeds. At higher speeds both measures stabilized: smoothness plateaued and the modular set expanded to four synergies, consistent with a more stable rhythmic organization. This correspondence was not limited to the group-level: smoothness and synergy dimensionality tracked one another within every participant, indicating that the kinematic and neuromuscular changes represent parallel signatures of a common speed-dependent reorganization rather than a direct causal relationship between the two measures. This profile supports the interpretation that walking speed modulates the balance between discrete- and rhythmic-like control.

More broadly, these findings support the utility of the dynamic-primitives framework for interpreting speed-dependent locomotor organization, with potential implications for motor learning^41^ and rehabilitation.^16^ Hogan and Sternad (2013) proposed that motor learning and recovery involve encoding dynamic primitive parameters to reconstruct movement patterns rather than reproducing specific behaviors, as seen in the progression of infant reaching from discrete submovements to continuous, rhythmic patterns.^42^^,^^43^ Similar patterns have been observed in stroke recovery, where early stereotyped submovements gradually become larger, fewer, and more integrated^44^^,^^45^^,^^46^ although it remains unclear whether similar mechanisms apply to lower limb locomotion.

### Limitations of the study

A few limitations should be considered. A few EMG channels were excluded in certain participants due to signal-quality criteria (e.g., noise, motion artifacts, electrode issues), which may influence absolute dimensionality estimates, although the main speed-dependent patterns remained evident. Treadmill walking differs from overground gait in sensory context and spatiotemporal characteristics; therefore, caution is warranted when generalizing absolute values. Nonetheless, the treadmill enabled systematic speed manipulation under controlled conditions. Our sample was predominantly female (12 females and 6 males). However, the present study was designed to characterize within-subject speed-dependent reorganization of locomotor control. Because each participant served as their own reference across the full speed range, stable participant characteristics, including sex-related differences, are inherently controlled by the within-subject design and are unlikely to bias the speed effects of interest. Moreover, the LDJ metric is normalized for movement duration and peak speed,^47^^,^^48^ which mitigates the influence of anthropometric differences. Consistent with this interpretation, a previous study reported low-to-negligible effects of sex on lower-limb muscle synergy number and reconstruction quality during walking.^29^ Therefore, although the sex distribution was unbalanced, it is unlikely to have influenced the primary outcomes of the present study.

A further consideration concerns the interpretation of the muscle synergies. Because muscle weightings and activation coefficients are estimated from the data, a successful reconstruction does not by itself establish that the recovered modules are neurally encoded primitives, and the extent to which synergies reflect neural control rather than task or biomechanical constraints remains debated.^49^^,^^50^ We therefore treat the present synergies as a description of the structure of the recorded muscle activity rather than as direct evidence of discrete neural modules. However, the speed-dependent changes in dimensionality were reproduced across two protocols (incremental and decremental) and two decompositions (subject- and group-level) and coincided with an independently quantified kinematic transition, none of which would be expected from the factorization procedure alone. Moreover, the merging analysis is a constrained, hypothesis-driven test in which the higher-speed synergies were held fixed as a basis and the lower-speed synergies were reconstructed only under pre-defined coefficient and similarity thresholds.^39^

Future work should replicate these analyses overground and with broader EMG coverage where feasible. Because movement smoothness can be estimated from wearable inertial sensors,^51^ these metrics offer a plausible route toward real-time monitoring of the discrete-rhythmic balance during adaptive locomotion and rehabilitation.

## Resource availability

### Lead contact

Requests for further information and resources should be directed to and will be fulfilled by the lead contact, Nadia Dominici (n.dominici@vu.nl).

### Materials availability

This study did not generate new unique reagents.

### Data and code availability

• All data have been deposited at Zenodo and are publicly available as of the date of publication at https://doi.org/10.5281/zenodo.22250456.
• All original code has been deposited at Zenodo and is publicly available at https://doi.org/10.5281/zenodo.20698517 as of the date of publication.
• Any additional information required to reanalyze the data reported in this paper is available from the lead contact upon request.

## Acknowledgments

This work was supported by 10.13039/501100000781European Research Council (ERC) under the European Union’s Horizon 2020 research and innovation program (grant agreement n: 715945 Learn2Walk), and by the Dutch Organization for Scientific Research (NWO) VIDI grant (grant agreement n: 016.156.346 FirSTeps), which supported the acquisition of the experimental instrumentation. We would like to thank Pascal Thom Sprenger for his support during data collection.

## Author contributions

Conceptualization, G.P., R.B., D.M., and N.D.; methodology, G.P. and N.D.; investigation, G.P. and N.D.; formal analysis: G.P.; writing – original draft, G.P.; writing – review & editing, R.B, D.M., and N.D.; funding acquisition, N.D.; resources, N.D.; supervision, R.B., D.M., and N.D.

## Declaration of interests

The authors declare no competing interests.

## STAR★Methods

### Key resources table

**Table:** 

REAGENT or RESOURCE	SOURCE	IDENTIFIER
**Software and algorithms**		

MATLAB 2024b	MathWorks	www.mathworks.com
Original analysis code	This paper	Zenodo: https://doi.org/10.5281/zenodo.20698517

**Deposited data**		

Raw kinematic and EMG data	This paper	Zenodo: https://doi.org/10.5281/zenodo.22250456.

### Experimental model and study participant details

#### Participants

18 healthy adults were recruited as participants for this study (12 female and 6 male, age 21.0 ± 2.5 years [mean ± SD], height 172.7 ± 8.8 cm, weight 65.7 ± 11.7 kg; see Table S1 for individual participant characteristics). *A priori* power analysis for a repeated-measures ANOVA (within-subjects design; power = 0.8, α = 0.05, effect size f = 0.25, one group) indicated that a minimum of 14 participants were required to detect statistically meaningful effects. We intentionally set the target recruitment above this threshold (*n* = 18) to account for inter-individual variability, potential attrition or unusable data due to technical issues. Exclusion criteria included the presence of neurological or musculoskeletal disorders, as well as recent, ongoing, or permanent injuries that may influence locomotor function and alter typical gait patterns.

The study was conducted in accordance with the guidelines of the Declaration of Helsinki. All procedures were approved by the Scientific and Ethical Review Board of the Faculty of Human Movement Sciences, Vrije Universiteit Amsterdam (VCWE-2025–119). Before the experiment, all participants read and signed a written informed consent form.

### Method details

#### Experimental design

Prior to the main experimental conditions, participants completed a familiarization phase on the treadmill to acclimate to walking at varying speeds. The main experimental protocol consisted of participants walking on an instrumented treadmill (N-Mill, 60 × 150 cm, Motek Medical B.V., Amsterdam, the Netherlands) under two conditions: incremental and decremental speed. Each participant performed one trial under each condition. In the incremental speed condition, participants started walking at 0.5 km/h, and the speed was increased in steps of 0.5 km/h until reaching 5 km/h. In the decremental speed condition, participants started walking at 5 km/h, and the speed was decreased in steps of 0.5 km/h until reaching 0.5 km/h. At each speed, participants completed 15 consecutive strides before the speed was changed. Short rest breaks were allowed when needed to minimize fatigue. The order of the incremental and decremental trials was randomized across participants.

#### Data acquisition

Kinematic data were recorded using a Vicon motion capture system (Vicon Vero v2.2, Oxford, UK) consisting of 10 infrared cameras placed around the treadmill and sampling at 100 Hz (Figure 1). A total of 19 infrared reflective markers of 14 mm diameter were positioned bilaterally on anatomical landmarks: heels, fifth metatarsophalangeal joints, lateral malleoli, lateral femur epicondyles, greater trochanters, iliac spinal crests, glenohumeral joints, humeral epicondyles, and ulnar styloids. An additional Vicon Vue video camera was positioned in the participants’ right sagittal plane at 100 Hz.

Muscle activity was recorded using a Wave Plus wireless EMG system with mini probes (Cometa, Milan, Italy) with Ag/AgCl electrodes (Ambu Blue Sensor). EMG signals were collected from 15 muscles bilaterally: tibialis anterior (TA), gastrocnemius lateralis (GL), gastrocnemius medialis (GM), soleus (SOL), peroneus longus (PER), rectus femoris (RF), vastus lateralis (VL), vastus medialis (VM), sartorius (SART), biceps femoris (BF), semitendinosus (SEM), tensor fasciae latae (TFL), gluteus maximus (GLM), gluteus medius (GMED) and erector spinae at the level of L2 (ES). These muscles were selected to represent the major functional groups involved in human walking and are widely used to characterize locomotor muscle synergies.^27^^,^^28^^,^^52^ The reliability of muscle synergies is influenced by the number of muscles included in the analysis, with stable structures emerging when more than ten muscles are considered.^53^ Because our analysis included 15 muscles per side (30 EMG channels in total), the number of recorded muscles exceeded this threshold, supporting the robustness of the extracted synergies. EMG signals were amplified with a gain of 1,000, and online band-pass filtered between 10 and 500 Hz before sampling at 2000 Hz.

In preparation, the participants’ skin was cleaned with alcohol at the locations where the electrodes were attached. Electrodes were attached to the skin over the muscle belly according to standard recommendations for minimizing crosstalk between adjacent muscles.^54^ Once all markers and electrodes were applied directly to the skin, participants' clothing was adjusted to ensure that they did not contact or interfere with either the markers or the electrodes. EMG and kinematics were simultaneously acquired and synchronized.

### Quantification and statistical analysis

#### Kinematic preprocessing and event detection

Data were processed using MATLAB custom script (2024b The MathWorks, Natick, MA, USA). Marker trajectories were extracted and low-pass filtered at 10 Hz (bidirectional 2nd-order Butterworth filter). Heel-strike (HS) and toe-off (TO) events were detected for both limbs using peak-based distance metrics^55^ and subsequently verified using an interactive GUI that allowed manual inspection and correction of gait events to ensure accuracy. HS and TO events were used to compute spatiotemporal gait parameters, including step time and stride time (from successive HS), cadence (steps/min), step and stride length, and stance, swing, and double-support durations and their percentages of the gait cycle for each limb. Treadmill speed was estimated from the anterior-posterior heel displacement between consecutive gait events and was used to segment each trial into constant-speed blocks.

#### Smoothness analysis

To capture inter-limb gait dynamics, we analyzed a relative ankle kinematic variable defined as the antero-posterior displacement between the right and left ankles (lateral malleoli marker).^25^^,^^56^ The discrete and rhythmic dynamics have been proposed to reflect a general organizational principle of motor control independent of any effector.^5^^,^^9^^,^^10^ Because locomotion is an inherently bilateral, alternating behavior, the functionally relevant oscillator in gait is the alternating motion of the two legs. The antero-posterior right-left ankle displacement provides a direct, low-dimensional descriptor of this inter-limb oscillation. Moreover, the same variable was used by Moura Coelho et al.^25^ to derive discrete and rhythmic locomotor biomarkers, ensuring direct methodological comparability with the previous locomotor application of the discrete-rhythmic framework. The right-left ankle displacement signal was demeaned and segmented between successive zero-crossings, with each segment defined by two zero-crossings sharing the same slope sign. Movement smoothness was then quantified for each segment using two complementary smoothness metrics: Log Dimensionless Jerk (LDJ) and Spectral Arc Length (SPARC).^47^^,^^48^^,^^51^ Lower smoothness values were taken to reflect a less continuous and more segmented movement pattern, whereas higher smoothness values were taken to indicate more continuous rhythmic dynamics.

Logarithmic Dimensionless Jerk (LDJ) is a time-domain metric that normalizes integrated squared jerk for movement duration and amplitude, making it largely invariant to scaling and enabling comparisons across conditions.^47^^,^^48^^,^^57^ LDJ was computed as:

(Equation 1) $$\eta_{LDJ}=-\ln\left(\left(\frac{{\left(t_{2}-t_{1}\right)}^{3}}{v_{peak}^{2}}\right)\int_{t_{1}}^{t_{2}}{\left|\frac{d^{3}x}{dt^{3}}\right|}^{2}dt\right)$$

LDJ values are negative, with values closer to zero (i.e., less negative) indicating smoother movements.^47^^,^^57^^,^^58^

Spectral Arc Length (SPARC) is a frequency-domain metric that quantifies smoothness from the spectral complexity of the speed profile, and has been shown to be robust to measurement noise and less sensitive to scaling effects than jerk-based measures.^47^^,^^48^^,^^51^ SPARC was computed as:

$$\eta_{SPARC}=-\int_{0}^{\omega_{c}}\sqrt{{\left(\frac{1}{\omega_{c}}\right)}^{2}+{\left(\frac{d\hat{V}\left(\omega \right)}{d\omega }\right)}^{2}}d\omega$$

(Equation 2) $$\hat{V}\left(\omega \right)=\frac{V\left(\omega \right)}{V\left(0\right)}$$

where ω_c is the upper frequency limit of integration, V(ω) is the magnitude spectrum of the speed profile, and V^(ω) is the spectrum normalized by its value at zero frequency. As with LDJ, SPARC values are negative, with values closer to zero indicating smoother movements.^47^^,^^48^ The two metrics were interpreted jointly to offset the respective limitations of time- and frequency-domain approaches.

#### EMG preprocessing

EMG data were visually inspected to identify artifacts, and corrupted data segments were excluded from further analysis. On average, 3.7 ± 2.0 EMG channels were excluded per participant. This corresponds to approximately 12% of the 30 bilateral channels, leaving the retained set well above the 10-muscles threshold for stable synergy extraction. Exclusions were applied at the whole-trial level, so the retained channel set was constant across all speed segments within a trial, and NNMF was performed on each participant’s available channels. The raw EMG was high-pass filtered at 30 Hz (bidirectional 4th-order Butterworth filter) to suppress low-frequency movement artifacts. Power-line noise was removed using a series of notch filters at 50, 100, and 150 Hz (bidirectional 4th-order Butterworth filter). In addition, to remove artifacts generated by the wireless accelerometer embedded in the EMG sensors a narrow band-stop filter was applied around 142 ± 2 Hz. The cleaned signal was then rectified and low-pass filtered at 10 Hz (bidirectional 4th-order Butterworth) to extract the linear envelope.

Right heel-strike events were used to segment the continuous EMG signal into individual gait cycles. Each gait cycle was time-normalized to 200 samples and amplitude-normalized to the peak EMG amplitude across the full trial.

For each stride, treadmill belt speed was estimated as the anterior-posterior velocity of the heel marker during the stance phase, reflecting the period of direct foot-belt contact. Speed-transition events identified from the kinematic segmentation were used to define the boundaries of speed-based EMG segments.

#### EMG synergy extraction

Muscle synergies were extracted from the EMG data using non-negative matrix factorization (NNMF), a linear decomposition technique that approximates the EMG data matrix as the product of two non-negative matrices representing time-invariant muscle weightings and time-varying activation profiles.^26^^,^^59^ Specifically, the EMG matrix $M\in R_{+}^{m\times n}$ (with m muscles and n time samples) was factorized as:

(Equation 3) M=W·H+E

where $W\in R_{+}^{m\times k}$ contains the synergy weight vectors (spatial modules or muscles weightings), $H\in R_{+}^{k\times n}$ contains the corresponding activation coefficients (temporal profiles or activation patterns), k is the number of synergies, and E is the residual error matrix. Equivalently, the EMG data can be expressed as a non-negative linear combination of k synergies:

(Equation 4) $$M\approx \sum_{i=1}^{k}w_{i}\cdot h_{i},w_{i}\geq 0,h_{i}\geq 0$$

Synergy analysis was performed separately for incremental and decremental conditions, and independently within each speed segment using two parallel NNMF pipelines: a subject-level and a group-level approach. At the subject level, EMG data were organized into speed-defined segments and time-normalized over the gait cycle; within each speed-segment, the cycle-averaged EMG matrix (mean across gait cycles) was factorized into Wand H. The number of synergies k was varied from 1 to 10; for each speed segment, NNMF was run 100 times with random initializations, with a maximum of 1,000 iterations per run, and the solution yielding the lowest reconstruction error was retained. Factorization was performed using the multiplicative update algorithm. Reconstruction quality was quantified as variance accounted for (VAF), computed as VAF = 1− SSE/SST, where SSE is the sum of squared reconstruction errors and SST is the total sum of squares of the original EMG data. The final number of synergies was selected using the 90% VAF criterion (smallest k such that VAF ≥0.90).^28^^,^^30^^,^^40^^,^^53^^,^^60^ This threshold is a widely adopted criterion and balances reconstruction fidelity against the over-fitting and noise-specific variance.

In parallel, at the group level, a segment-wise group EMG matrix was constructed by averaging each subject’s cycle-averaged EMG profile within each speed segment, resulting in a single $M\in R_{+}^{m\times n}$ matrix for each segment and condition. The same NNMF procedure (k = 1–10; 100 runs per k; 1000 iterations, 90% VAF criterion) was applied to obtain group-representative synergy structures. Because NNMF components are not inherently ordered, synergies were first ordered by the timing of their peak activation in H in a reference (“leader”) segment (5 km/h) and then matched across segments using a similarity criterion to ensure consistent labeling across speeds.

#### Merging analysis

To examine whether changes in synergy dimensionality across walking speeds could be explained by merging of the modules, a reconstruction-based procedure was implemented following Cheung et al., (2020). In this approach, synergy merging refers to the process by which distinct synergies active at higher speeds are subsumed into fewer synergies at lower speeds; under this model, each slower-speed synergy can be approximated as a non-negative linear combination of faster-speed synergies. Evidence for merging was therefore inferred when synergies from the faster segment successfully reconstructed those from the slower speed-segment, consistent with a reduction in synergy number driven by the combining of modules across speeds.

For each trial type, the group-averaged synergy weight matrices (W) estimated within each speed segment were analyzed. Merging was evaluated across adjacent speed segments in the direction faster → slower, such that synergies from the faster segment were used as basis vectors to reconstruct synergies from the slower segment. Prior to fitting, all synergy weight vectors were normalized to unit length.^39^

For each target synergy $w_{i}^{A}$ in the slower speed-segment A (segA), non-negative coefficients *m*_*ik*_ were estimated to best reconstructed the target from the faster speed-segment synergies $w_{k}^{B}$ in segment B (segB) using non-negative least squares (MATLAB function lsqnonneg). The reconstruction model was:

(Equation 5) $$w_{i}^{A}\approx \sum_{k=1}^{N_{B}}m_{ik}w_{k}^{B},m_{ik}\geq 0$$

To retain only meaningful contributors, a threshold of m_ik_ ≥ 0.20 was applied to the estimated coefficients, as defined in.^39^

A target synergy was classified as merged when it was reconstructed by at least two contributing synergies above this threshold and the cosine similarity between the target and its reconstruction was at least 0.80.^39^ For each transition, merging was summarized as the count and proportion of target synergies in the slower speed-segment meeting these criteria.

#### Statistical analysis

In order to quantify whether smoothness parameters systematically changed across speed-defined segments within individual trials, a within-trial repeated-measures analysis was conducted separately for each subject and condition. A repeated-measures ANOVA with segment as the within-trial factor was used to test for an overall speed segment effect. Where a significant effect was found, pairwise post-hoc comparisons between segments were extracted from the fitted repeated-measures model, with *p*-values adjusted for multiple comparisons using the Holm adjustment, which was preferred for its balance between control of familywise error rate and statistical power.

In the present study, the transition was interpreted as the speed range over which smoothness measures showed their clearest reorganization across speed segments, rather than as a single threshold.

To examine whether the number of extracted muscle synergies systematically changed across speed-defined segments, a within-subject repeated-measures analysis was conducted separately for the incremental and decremental conditions. For each subject, the number of synergies identified per speed segment served as the unit of observation. A one-factor repeated-measures ANOVA with speed as the within-subject factor was used to test for an overall speed effect. Pairwise post-hoc comparisons between speed levels were obtained from the fitted model, with *p*-values adjusted for multiple comparisons using the Holm adjustment.

To visualize the consistency of speed-related effects across participants, pairwise post-hoc comparisons were summarized as heatmaps. Because the repeated-measures ANOVA and post-hoc contrasts were computed separately for each participant and condition, each participant contributed a binary outcome, significant or non-significant, for every pairwise speed comparison. Pooling these binary outcomes across participants yields, for each speed pair, the percentage of participants for whom that comparison reached significance (Holm-adjusted *p* < 0.05), represented by a single heatmap cell (0–100%).

To quantify the association between kinematic smoothness and muscle synergy dimensionality at the individual level, a within-subject correlation analysis was performed. For each participant and condition, the mean value for each smoothness metric (calculated separately for LDJ and SPARC) was paired with the number of synergies extracted from the corresponding segment. The association was quantified using Spearman’s rank correlation coefficient (ρ) within each participant and summarized across participants as the median and interquartile range (IQR) of the per-subject coefficients, together with the proportion of participants showing a significant association within each condition (*p* < 0.05).

## Supplemental information

Document S1. Figure S1 and Tables S1–S6

Table S7. Per-participant pairwise post hoc contrasts of kinematic smoothness

Table S8. Pairwise post hoc contrasts of synergy number in incremental condition

Table S9. Pairwise post hoc contrasts of synergy number in decremental condition
