# Supplementary material for: Speed-driven transitions between discrete and rhythmic dynamics in walking revealed by kinematic smoothness and muscle synergies
Source: iScience. 2026 Oct 7;29(11):117779. doi: 10.1016/j.isci.2026.117779 (PMC13676916; doi:10.1016/j.isci.2026.117779)
Supplement: Supplementary file 1 [file mmc1.pdf]

iScience, Volume *29*

Supplemental information

Speed-driven transitions between discrete and rhythmic dynamics in walking revealed by kinematic smoothness and muscle synergies

Giulia Panconi, Diego Minciacchi, Riccardo Bravi, and Nadia Dominici

Figure S1. Correlation between kinematic smoothness and muscle synergy dimensionality across walking speeds. Number of extracted muscle synergies plotted against LDJ (A, C) and SPARC (B, D), in the incremental (A, B) and decremental (C, D) conditions. Each point represents one participant at one speed segment, color-coded by walking speed (0.5–5.0 km/h; color bar). The dashed black line indicates the least-squares linear fit.

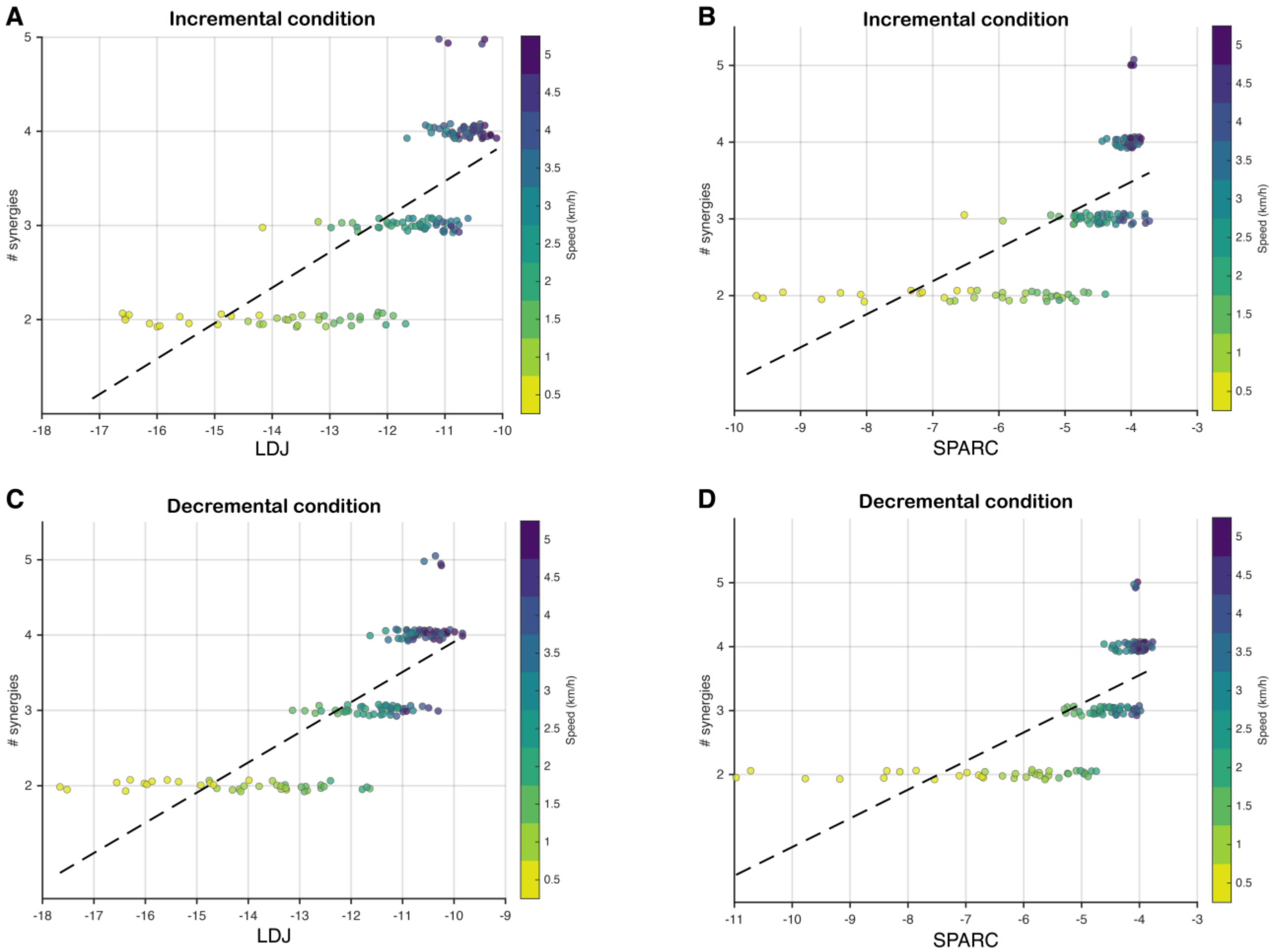

Table S1. Participant Characteristics

Subjects	Age (years)	Gender	Weight (kg)	Height (cm)
P1	21	F	64	168
P2	21	F	60	166
P3	23	M	80	190
P4	22	M	80	180
P5	27	F	48	160
P6	22	M	86	186
P7	22	M	72	179
P8	22	M	80	183
P9	18	F	72	182
P10	25	M	79	175
P11	22	F	55	167
P12	20	F	53	163
P13	19	F	54	166
P14	17	F	53	162
P15	19	F	66	171
P16	20	F	56	169
P17	19	F	59	165
P18	19	F	64	172

Table S2. Spatiotemporal gait parameters (mean ± SD across participants) per speed segment in incremental condition.

Parameter	0.5	1.0	1.5	2.0	2.5	3.0	3.5	4.0	4.5	5.0
Stride time (s)	4.00 ± 0.86	2.57 ± 0.30	2.00 ± 0.13	1.70 ± 0.10	1.50 ± 0.07	1.36 ± 0.05	1.26 ± 0.05	1.19 ± 0.04	1.13 ± 0.04	1.08 ± 0.04
Cadence (steps/min)	31.2 ± 4.6	48.5 ± 6.5	60.9 ± 4.6	71.0 ± 4.4	80.8 ± 4.1	88.6 ± 3.8	95.4 ± 3.7	101.6 ± 3.7	106.6 ± 4.2	111.4 ± 4.7
Stride length (cm)	56.4 ± 10.5	68.6 ± 8.6	80.4 ± 5.9	89.7 ± 5.9	98.7 ± 5.1	106.8 ± 4.7	115.1 ± 4.7	122.9 ± 4.8	131.1 ± 5.5	138.1 ± 5.9
Stance (% stride)	83.4 ± 2.1	78.0 ± 2.0	74.3 ± 1.6	72.5 ± 0.9	70.5 ± 1.2	69.5 ± 1.0	68.4 ± 1.0	67.8 ± 0.8	67.2 ± 0.7	66.5 ± 0.7
Swing (% stride)	16.7 ± 2.2	22.0 ± 2.0	25.7 ± 1.6	27.5 ± 0.9	29.5 ± 1.2	30.5 ± 1.0	31.6 ± 1.0	32.2 ± 0.8	32.8 ± 0.7	33.5 ± 0.7
Double support (% stride)	66.7 ± 4.4	56.1 ± 4.1	48.5 ± 3.5	45.1 ± 1.6	41.0 ± 2.3	39.1 ± 1.9	36.9 ± 1.9	35.6 ± 1.6	34.4 ± 1.4	33.1 ± 1.3

Table S3. Spatiotemporal gait parameters (mean ± SD across participants) per speed segment in decremental condition.

Parameter	0.5	1.0	1.5	2.0	2.5	3.0	3.5	4.0	4.5	5.0
Stride time (s)	4.14 ± 0.94	2.60 ± 0.32	2.03 ± 0.17	1.70 ± 0.10	1.51 ± 0.06	1.36 ± 0.05	1.26 ± 0.05	1.18 ± 0.05	1.11 ± 0.05	1.07 ± 0.04
Cadence (steps/min)	32.0 ± 5.5	48.3 ± 6.4	61.5 ± 7.9	71.0 ± 4.5	79.9 ± 3.6	88.3 ± 3.7	95.7 ± 3.7	101.9 ± 4.0	107.9 ± 4.5	112.6 ± 4.5
Stride length (cm)	57.1 ± 10.0	69.2 ± 7.7	80.0 ± 5.4	89.1 ± 5.3	98.4 ± 4.5	106.4 ± 4.0	114.6 ± 4.3	122.4 ± 4.6	129.6 ± 5.0	137.0 ± 5.5
Stance (% stride)	80.7 ± 10.4	75.8 ± 6.7	74.2 ± 1.2	71.8 ± 1.0	70.4 ± 0.8	69.3 ± 0.8	68.4 ± 0.7	67.8 ± 0.7	67.1 ± 0.7	66.4 ± 0.6
Swing (% stride)	19.3 ± 10.4	24.2 ± 6.7	25.8 ± 1.2	28.2 ± 1.0	29.6 ± 0.8	30.7 ± 0.8	31.6 ± 0.7	32.2 ± 0.7	32.9 ± 0.7	33.6 ± 0.6
Double support (% stride)	66.4 ± 5.7	55.0 ± 5.2	48.4 ± 2.5	43.6 ± 1.9	40.9 ± 1.6	38.7 ± 1.7	36.8 ± 1.3	35.6 ± 1.5	34.2 ± 1.3	32.9 ± 1.3

Table S4. Omnibus effect of speed on kinematic smoothness

Metric - condition	F	*p*
LDJ - Incremental	$F(9, 144) = 384.17$	5.69×10^{-96}
LDJ - Decremental	$F(9, 144) = 336.98$	4.68×10^{-92}
SPARC - Incremental	$F(9, 144) = 189.28$	3.70×10^{-75}
SPARC - Decremental	$F(9, 144) = 166.83$	1.49×10^{-71}

Table S5. Omnibus effect of speed on the number of muscle synergies

Condition	F	*p*
Incremental	$F(9, 144) = 85.49$	3.29×10^{-47}
Decremental	$F(9, 144) = 80.02$	8.63×10^{-48}

Table S6. Within-participant correlation between kinematic smoothness and synergy number

Metric - condition	Median ρ	IQR	Range	Significant	Pooled ρ (Fisher z) [95 % CI]
LDJ - Incremental	.921	.921-.944	.853-.969	100%	.928 [.894, .951]
LDJ - Decremental	.921	.881-.934	.696-.960	100%	.910 [.869, .938]
LDJ - Both combined	.909	.870-.926	.738-.936	100%	.897 [.869, .919]
SPARC - Incremental	.921	.921-.944	.853-.944	100%	.922 [.886, .947]
SPARC - Decremental	.921	.881-.934	.696-.960	100%	.910 [.869, .938]
SPARC - Both combined	.911	.866-.921	.776-.934	100%	.895 [.866, .918]
